# COX-2 gene rs689466 polymorphism and nicotine dependence among schizophrenia patients

**DOI:** 10.1192/j.eurpsy.2025.609

**Published:** 2025-08-26

**Authors:** S. Nadalin, M. Vilibić, V. Peitl, A. Silić, I. Ljoka, I. Majdandžić, P. Posavec, D. Karlović, A. Buretić-Tomljanović

**Affiliations:** 1 Department of Psychiatry, General Hospital “Dr. Josip Benčević”, Slavonski Brod; 2 School of Medicine, Catholic University of Croatia; 3 Department of Psychiatry, Sestre Milosrdnice University Hospital Center, Zagreb; 4 Department of Anesthesiology, Reanimatology and Intensive Care Medicine, General Hospital “Dr. Josip Benčević”, Slavonski Brod; 5 Faculty of Health Studies, University of Rijeka; 6 Department of Medical Biology and Genetics, Faculty of Medicine, University of Rijeka, Rijeka, Croatia

## Abstract

**Introduction:**

In addition to play a key role in the generation of the inflammatory response, cyclooxygenases (COXs) are involved in a variety of other physiological processes, including dopaminergic signaling, and the skin flush response to the water-soluble B vitamin niacin. Patients with schizophrenia frequently exhibit abnormal signaling of dopamine and other neurotransmitters and attenuated niacin skin flush response. Clinical studies reported beneficial effects of selective cyclooxygenase-2 (COX-2) inhibitors on the simptom expression of schizophrenia, as measured by the Positive and Negative Syndrome Scale (PANSS). Several studies investigated potential correlations between functional rs689466 polymorphism (A/G polymorphism) of the COX-2 gene and etiology of schizophrenia, but the results have not been consistent. While we did not find evidence that the COX-2 polymorphism was associated with an elevated risk of schizophrenia risk in a Croatian population, we observed that the COX-2 polymorphism contributes to attenuated niacin skin flushing in patients with schizophrenia.

**Objectives:**

To the best of our knowledge, no studies have investigated the potential associations between any COX-2 gene polymorphisms and the etiology of nicotine dependence. Based on the elevated smoking rate observed consistently in patients with schizophrenia and the possible relevance of COX-2 genes in schizophrenia and nicotine dependence via dopaminergic signaling, we investigated whether the risk of nicotine dependence among patients with schizophrenia is associated with the COX-2 gene rs689466 polymorphism. Given evidence suggesting that smoking influences the severity of schizophrenia, we also hypothesized that an interaction between smoking and the COX-2 polymorphism may contribute to the age of schizophrenia onset and/or PANSS psychopathology.

**Methods:**

Polymerase chain reaction analysis/restriction fragment length polymorphism analysis was used to genotype 190 chronically ill schizophrenia patients treated with antipsychotic medications (104 males/86 females).

**Results:**

There were no significant differences in the distribution of COX-2 genotypes and alleles in male or female schizophrenia patients who were stratified based on their smoking status and no COX-2 genotype-smoking interaction on PANSS psychopathology (P > 0.05). We revealed a significant COX-2 genotype-smoking interaction on the time of disease onset among female patients (P < 0.05). An earlier onset, observed for female smokers carrying the G-allele in their COX-2 genotypes (COX-2-GG homozygous and COX-2-AG heterozygous), in comparison to nonsmoking COX-2-G allele carriers, contributed mostly to this finding.

**Conclusions:**

Our results indicate no COX-2 gene polymorphism’s effect on the risk of nicotine dependence, but they suggest that COX-2 genotype-smoking interaction might be of relevance in disease onset, in a gender-specific fashion.

**Disclosure of Interest:**

None Declared

